# Preventive effect of pentoxifylline on contrast-induced acute kidney injury in hypercholesterolemic rats

**DOI:** 10.3892/etm.2014.2132

**Published:** 2014-12-15

**Authors:** SHI-KUN YANG, SHAO-BIN DUAN, PENG PAN, XIANG-QING XU, NA LIU, JUN XU

**Affiliations:** Department of Nephrology, Second Xiangya Hospital of Central South University; Nephrology Institute of Central South University; Centre of Kidney Disease and Dialysis, Changsha, Hunan 410011, P.R. China

**Keywords:** pentoxifylline, low-osmolar contrast media, acute kidney injury, oxidative stress, nicotinamide adenine dinucleotide phosphate oxidase

## Abstract

Oxidative stress is an important mechanism of contrast-induced acute kidney injury (CIAKI). The optimal strategy to prevent CIAKI remains unclear. The aim of the present study was to assess the effect of pentoxifylline, a nonspecific phosphodiesterase inhibitor, on the prevention of CIAKI. A total of 32 healthy male Sprague-Dawley rats were randomly divided into normal dietary group (NN; n=8) and a high cholesterol-supplemented dietary group (HN; 4% cholesterol and 1% cholic acid; n=24). At the end of eight weeks, the rats in the high cholesterol diet group were randomly divided into three subgroups (n=8 in each group). CIAKI was induced in two of the subgroups via intravenous injection of the radiocontrast media iohexol (10 ml/kg). Pentoxifylline (50 mg/kg) was administered to one of the iohexol-treated groups via intraperitoneal injection 12 h prior to and following contrast media (CM) injection. Kidney function parameters and oxidative stress markers were then measured. The renal pathological changes were evaluated using hematoxylin and eosin staining and scored semi-quantitatively. In iohexol-injected rats, serum creatinine (Scr), renal pathological scores, renal malondialdehyde (MDA) content, renal NADPH oxidase activity, fractional excretion of sodium (FENa%) and fractional excretion of potassium (FEK%) were significantly increased (P<0.01). The Scr, histologic scores, renal MDA content, NADPH oxidase activity, FENa% and FEK% in the rats treated with pentoxifylline prior to iohexol were observed to be reduced compared with those in rats treated with iohexol alone (P<0.01). This suggests that pentoxifylline significantly attenuates renal injuries, including tubular necrosis and proteinaceous casts induced by CM. It may be concluded that pentoxifylline protected the renal tissue from the nephrotoxicity induced by low-osmolar CM via an antioxidant effect.

## Introduction

Contrast-induced acute kidney injury (CIAKI) is a serious clinical complication, associated with increased use of iodinated contrast media (CM) in diagnostic and interventional procedures. It is now the third most important cause of hospital-acquired acute kidney injury and accounts for 12% of all cases (1). Although the use of low-osmolar CM (LOCM) reduces the risk of CIAKI, the incidence of CIAKI remains high following intravascular administration of LOCM in high-risk patients with renal insufficiency (2).

The pathogenic mechanism of CIAKI is multifactorial, and previous studies have shown that it is mainly associated with oxidative stress, renal ischemia and direct nephrotoxicity (3). In addition, a series of endogenous vasoactive substances, including renin and angiotensin II, have an important role in the pathogenesis of CIAKI (4). NADPH oxidase is the major source of reactive oxygen species (ROS) and is significant in cisplatin- or cyclosporine-induced acute kidney injury (5). In previous studies, we demonstrated that NADPH oxidase was a pivotal contributor to oxidative stress damage in CIAKI (6,7). These results suggest that NADPH oxidase might be a novel target for the development of drugs against CIAKI.

Pentoxifylline, a nonspecific phosphodiesterase inhibitor, is a methylxanthine derivative with multiple hematologic properties (8). It has been demonstrated that pentoxifylline may be used as a renoprotective agent against a number of nephrotoxic drugs, including cyclosporine or cisplatin in clinical and animal studies (9), and a recent study performed by Firouzi *et al* (10) concluded that the prophylactic oral use of pentoxifylline may be recommended for the prevention of CIAKI. However, the mechanism for its protective effect has yet to be elucidated. Therefore, the aim of the present study was to further evaluate the protective effect of pentoxifylline on CIAKI in rats with hypercholesterolemia and the underlying mechanisms.

## Materials and methods

### Reagents and animals

Low-osmolar iodinated contrast medium, iohexol (Amersham Pharmaceutical Co., Ltd, Shanghai, China), was diluted to 300 mg I/ml with distilled water. Cholesterol and cholic acid were obtained from DingGuo Biotechnology Co., Ltd. (Beijing, China) and the NADPH oxidase assay kit was purchased from Jiemei Gene Medicine Technology Co., Ltd. (Shanghai, China). The MDA and SOD detection kits were purchased from Jiancheng Bioengineering Institute (Nanjing, China). Pentoxifylline was obtained from Northeast Pharmaceutical Group Shenyang No.1 Pharmaceutical Co., Ltd. (Shenyang, China).

A total of 32 healthy male Sprague-Dawley rats, weighing 160–180 g, with negative tests for urine protein and glucose, were provided by the Second Xiangya Hospital Animal Center (Changsha, China). The rats were randomly divided into the normal diet group (NN; n=8) and high cholesterol-supplemented dietary group (HN; 4% cholesterol and 1% cholic acid; n=24) (11). After eight weeks, the rats in the HN group were randomly divided into three subgroups (n=8 in each group): the high cholesterol diet group (HN), the high cholesterol plus LOCM iohexol group (HL) and the pentoxifylline protective group (high cholesterol plus iohexol plus pentoxifylline; HLP). All experiments were approved by the Medical Science Animal Care Committee of the Central South University (Changsha, Hunan, China).

### Experimental treatment

At the end of the eight weeks of feeding, the eight rats in each group were given a tail vein injection of either iohexol (10 ml/kg; HL and HLP groups) or an equivalent volume of normal saline (HN and NN groups) over 2 min. The rats in the HLP group were injected with pentoxifylline (50 mg/kg) into the peritoneal cavity, 12 h prior to and following CM injection. Rats from the NN, HN and HL groups were administered an equal volume of normal saline. Urine samples and blood samples were obtained prior to and 48 h following the injection of CM to determine the level of serum creatinine (SCr), triglyceride (TG), cholesterol (CHOL), urine creatinine, sodium and potassium, using an automated biochemical analyzer (Olympus AU100, Tokyo, Japan). In addition, creatinine clearance (Ccr), fractional excretion of sodium (FENa%) and potassium (FEK%) were calculated as previously described (12).

Once the blood samples were obtained, the rats were sacrificed by cervical dislocation. The right kidney tissue was homogenized to measure the levels of malondialdehyde (MDA), superoxide dismutase (SOD) and nicotinamide adenine dinucleotide phosphate-oxidase (NADPH oxidase) activity using commercial kits. The total protein content of renal tissue was then measured using the Coomassie brilliant blue method.

### Renal tissue MDA, SOD and NADPH oxidase assay

In brief, 200 mg renal cortex was washed in ice-cold saline in tubes, sectioned into small pieces and homogenized in ice-cold saline homogenization buffer in a 1:9 ratio (w:v). The homogenate was centrifuged at 2971 × g for 10 min at 4°C. The supernatant was separated and analyzed for total protein content and SOD and NADPH oxidase activity. The protein concentration of the homogenate was determined using the BCA assay method. The results were expressed as nmmol/mg protein (MDA) and U/mg protein (SOD and NADPH oxidase).

### Renal histopathological evaluation

The upper halves of the left kidney were cut into sections at 4 μm and stained using hematoxylin and eosin for histopathological evaluation under a light microscope (Leica DMI-3000B; Leica Microsystems AG, Wetzlar, Germany). Evaluations were performed and scored semi-quantitatively in a blinded manner using an arbitrary scale (6). The calculation was determined at a ×200 magnification in 10 fields for each biopsy. Tubular injuries were graded as follows: 0, no tubular injury; 1, <25% of tubules injured; 2, between 25 and 50% of tubules injured; 3, between 51 and 75% of tubules injured; 4, >76% of tubules injured (7).

### Statistical analysis

Statistical analyses were performed using SPSS software, version 16.0 (SPSS, Inc., Chicago, IL, USA). The results are expressed as the mean ± standard deviation. Data were analyzed using one-way analysis of variance, and the two-groups comparison among multiple samples was performed using the Fisher Least Significant Difference test. P<0.05 was considered to indicate a statistically significant difference.

## Results

### Baseline characteristics in different groups

Body weight, serum TG, CHOL, Scr, Ccr, FENa% and FEK% values were analyzed in the four groups at baseline. As shown in Table I, there were no significant differences in the baseline characteristics of Scr, Ccr, FENa% and FEK% (P>0.05). In the rats fed a high cholesterol diet, the levels of serum CHOL were higher compared with those in rats fed a normal diet (P<0.05). All rats tolerated the treatment well, and survived until the end of the experiment.

### Effect of pentoxifylline on renal function parameters

In CM-treated groups, rats showed marked increases in Scr, FENa% and FEK% (P<0.01), and a decline in Ccr (P<0.01) following iohexol administration, compared with those in control animals. However, the CM-treated rats that underwent treatment with pentoxifylline showed significantly decreased values of Scr, FENa% and FEK% compared with those in the rats treated with CM alone (P<0.05; Table II).

### Effect of pentoxifylline on renal oxidative stress parameters and NADPH oxidase activity

As shown in Table III, in CM-treated rats, a trend towards higher values of renal MDA levels and NADPH oxidase activity was observed compared with control animals (P<0.01), while the renal SOD activity was lower in the CM-treated rats than in the control rats (P<0.01). However, in the rats treated with pentoxifylline and iohexol, the renal NADPH oxidase activity and MDA levels were significantly lower compared with those in the rats treated with iohexol alone (P<0.01), while a significant increase in the activity of renal SOD was observed (P<0.01).

### Histomorphological comparison of renal injury

**Figure 1:**
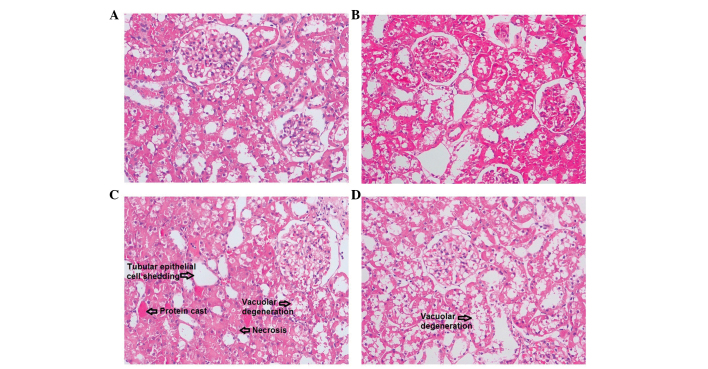
Representative renal histomorphological changes in the (A) NN, normal diet group, (B) HN, high cholesterol diet group, (C) HL, high cholesterol plus low-osmolar contrast media iohexol group and (D) HLP, high cholesterol plus iohexol plus pentoxifylline group. (C) In iohexol-injected rats, tubular epithelial cell shedding and basement membrane nudity, vacuolar degeneration of tubular epithelial cells, protein cast, tubular dilation, loss of tubular brush border, and necrosis of partial tubular epithelial cells were observed. However, in (D) the rats treated with pentoxifylline and iohexol, only renal tubular epithelial cell degeneration was observed. Hematoxylin and eosin staining, original magnification, ×200.

**Figure 2:**
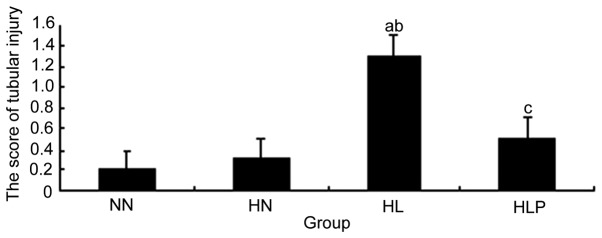
Comparison of tubular injury scores in each group following contrast media injection. The scores of tubular injury in iohexol-injected rats were significantly higher compared with those in control animals, while the scores of tubular injury in the rats treated with pentoxifylline and iohexol were significantly lower compared with those in rats treated with iohexol alone. ^a^P<0.01, versus the NN group; ^b^P<0.01, versus the HN group; ^c^P<0.01, versus the HL group. NN, normal diet group; HN, high cholesterol diet group; HL, high cholesterol plus low-osmolar contrast media iohexol group; HLP, high cholesterol plus iohexol plus pentoxifylline group.

As shown in Fig. 1, tubular epithelial cell shedding and basement membrane nudity, vacuolar degeneration of tubular epithelial cells, protein cast, tubular dilation, loss of tubular brush border, and necrosis of partial tubular epithelial cells was observed in CM-injected rats. However, in the pentoxifylline-injected rats, only renal tubular epithelial cell degeneration was observed. As shown in Fig. 2, light microscopic examination and semi-quantitative analysis demonstrated that the tubular pathological scores in the HL group were significantly higher compared with those of the control animals (P<0.01). Pretreatment with pentoxifylline was observed to significantly attenuate the development of these lesions, as the tubular pathological scores of the rats treated with iohexol alone were significantly higher compared with those of iohexol-treated rats that were pretreated with pentoxifylline (P<0.01).

## Discussion

In the present study, it was investigated whether pentoxifylline has protective effect against CIAKI. Pentoxifylline is known to have antioxidant properties. It is also cost-effective, readily available and is currently being used clinically for chronic kidney disease (13). The results from the present study have further demonstrated that pentoxifylline may also protect against CIAKI in rats with hypercholesterolemia, which may be due to an antioxidant action.

The pathological mechanism of CIAKI remains unclear. In a previous study we demonstrated that high-osmolality CM induces cultured human renal tubular epithelial cell apoptosis, while LOCM does not induce tubular epithelial cell apoptosis (14). In addition, clinical studies have demonstrated a reduction in contrast nephropathy with the introduction of LOCM (15). However, the cytotoxicity of radiocontrast agents has been shown to correlate with not only hyperosmolality, but also iodine concentration (14). The osmotic pressures of high-osmolality CM (>1,500 mOsm/kg) and LOCM (550–850 mOsm/kg) are higher than those of plasma (280–310 mOsm/kg). LOCM is an important risk factor for radiocontrast nephrotoxicity, and in the present study, a CIAKI model was successfully established using the LOCM iohexol in rats with hypercholesterolemia. This was consistent with the study by Yang *et al* (12), which observed that long-term hypercholesterolemia diet and LOCM appeared to be risk factors for CIAKI (12).

A recent study demonstrated that oxidative stress has an important role in the pathogenesis of CIAKI (3). NADPH oxidases represent a class of hetero-oligomeric enzymes whose primary function is the generation of ROS. In addition, NADPH oxidase has been shown to have an important role in cisplatin or cyclosporine-induced acute kidney injury (5). Furthermore, a recently study by Ahmad *et al* (16) demonstrated that the NADPH-oxidase inhibitor apocynin attenuates the degree of contrast-induced nephropathy in diabetic rats (16). The present study demonstrated that in the iohexol-injected rats, the levels of Scr, renal tissue MDA content and NADPH oxidase activity were significantly increased. In addition, in the iohexol-treated animals compared with the control animals, the tubular pathological injury scores were found to be higher, while the Ccr and renal SOD activity decreased significantly. Furthermore, it was demonstrated that NADPH oxidase and oxidative stress appear to have an important role in CIAKI.

There is currently no accepted method for the prevention of CIAKI, apart from the importance of hydration and avoidance of hypovolemia prior to exposure to CM (17). Studies have shown that the formation of ROS is increased in the kidney following the administration of CM, which has an important role in the development of CIAKI (18). In accordance with previous studies, the results from the present study showed that iohexol administration significantly increased renal oxidative stress, suggesting a protective effect of ROS scavenging in CIAKI.

Pentoxifylline, an inhibitor of phosphodiesterase, was first considered in the treatment of peripheral vascular diseases (19). It is known to have several pharmacological effects, including inhibition of free radical production, stimulation of the biosynthesis of renal vasodilator prostaglandins, and improvement in oxygen delivery to ischemic tissues (20). Additionally, it has been shown that pentoxifylline is able to inhibit the formation of free radicals by inhibiting NADPH oxidase activity in neutrophils (21). The results from animal and clinical studies suggest that pentoxifylline may prevent renal cell injuries induced by glycerol, cisplatin or cyclosporine (9,22). A recent study by Firouzi *et al* (10) indicated that the prophylactic oral use of pentoxifylline may be recommended for CIAKI prevention; however, the mechanisms for this protective effect have yet to be elucidated.

The results from the present study showed that pretreatment with pentoxifylline prior to iohexol infusion may reduce the SCr and the renal MDA content and NADPH oxidase activity. In the pentoxifylline treatment group, the tubular pathological changes associated with iohexol were found to be reduced, while the levels of renal SOD activity were increased. This indicates that pentoxifylline has a protective effect against the nephrotoxicity induced by iohexol in renal tissue, due to an antioxidant action.

There are several potential limitations of the present study that should be addressed. Firstly, pentoxifylline has been found to have novel biochemical effects, including the alleviation of renal vasoconstriction (23), reduction of oxidative stress and modulation of inflammatory responses (24). These properties may also be used to prevent CIAKI as proposed by Roozbeh *et al* (8). The main limitation of this study is that the mechanism of the anti-oxidation effects of pentoxifylline was analyzed only in CIAKI. Secondly, only tubular injury scores were measured to indicate the degree of tubular injury, and TUNEL immunostaining and analysis of urine tubular injury biomarkers (for example, NGAL) was not performed. However, despite these limitations, the results from the present study remain valid.

In conclusion, the results from the present study demonstrated that pentoxifylline is able to protect the renal tissue from the nephrotoxicity induced by CM in rats with hypercholesterolemia due to an antioxidant action. However, further clinical trials in patients with CIAKI are required in order to determine its exact role.

## Figures and Tables

**Table I:** Comparison of biochemical indicators in each group prior to contrast media injection (mean±standard deviation; n=8).

Group	BW (g)	Scr (μmol/l)	TG (mmol/l)	CHOL (mmol/l)	Ccr (ml/min)	FENa%	FEK%
NN	321.26±85.27	32.13±2.96	0.32±0.17	1.35±0.18	0.41±0.05	1.36±0.42	71.24±7.08
HN	334.30±72.67	35.50±2.44	0.39±0.18	2.71±0.14^a^	0.39±0.04	1.48±0.38	75.00±6.63
HL	317.54±86.80	34.61±2.49	0.41±0.21	2.86±0.22^a^	0.38±0.03	1.56±0.31	74.30±6.96
HLP	321.68±79.46	34.92±2.73	0.48±0.20	2.62±0.25^a^	0.38±0.02	1.61±0.47	73.84±5.84

a P<0.05, versus NN group.

NN, normal diet group; HN, high cholesterol diet group; HL, high cholesterol plus low-osmolar contrast media iohexol group; HLP, high cholesterol plus iohexol plus pentoxifylline group; BW, body weight; Scr, serum creatinine; CHOL, cholesterol; Ccr, creatinine clearance rate; FEK%, fractional excretion of potassium; FENa%, fractional excretion of sodium.

**Table II:** Changes in renal function indicators in each group 48 h following contrast media injection (mean±standard deviation; n=8).

Group	Scr (μmol/l)	Ccr (ml/min/100 g)	FENa%	FEK%
NN	32.95±2.14	0.42±0.04	1.28±0.25	70.61±5.09
HN	35.62±1.81	0.40±0.03	1.55±0.41	75.29±4.13
HL	46.69±2.91^a^	0.29±0.02^a^	3.95±0.15^a^	98.25±4.08^a^
HLP	36.16±2.06^b^	0.39±0.03^b^	1.78±0.16^b^	77.12±5.97^b^

a P<0.01, versus the HN group;

b P<0.01, versus the HL group.

NN, normal diet group; HN, high cholesterol diet group; HL, high cholesterol plus low-osmolar contrast media iohexol group; HLP, high cholesterol plus iohexol plus pentoxifylline group; Scr, serum creatinine; Ccr, creatinine clearance rate; FEK%, fractional excretion of potassium; FENa%, fractional excretion of sodium.

**Table III:** Comparison of renal oxidative stress parameters following contrast media injection (mean±standard deviation; n=8).

Group	MDA (nmmol/mg protein)	SOD (U/mg protein)	NADPH oxidase (U/mg protein)
NN	3.43±0.47	413.03±23.28	14.95±5.12
HN	4.21±0.75	394.67±43.62	21.26±8.26^a^
HL	8.46±0.92^a^,^b^	317.01±47.36^a^,^b^	82.42±31.18^a^,^b^
HLP	5.27±0.48^c^	422.32±41.50^c^	26.59±7.56^c^

a P<0.01, versus the NN group;

b P<0.01, versus the HN group;

c P<0.01, versus the HL group.

NN, normal diet group; HN, high cholesterol diet group; HL, high cholesterol plus low-osmolar contrast media iohexol group; HLP, high cholesterol plus iohexol plus pentoxifylline group; NADPH oxidase, nicotinamide adenine dinucleotide phosphate-oxidase; MDA, malondialdehyde; SOD, superoxide dismutase.
